# Predictive Monitoring–Impact in Acute Care Cardiology Trial (PM-IMPACCT): Protocol for a Randomized Controlled Trial

**DOI:** 10.2196/29631

**Published:** 2021-07-02

**Authors:** Jessica Keim-Malpass, Sarah J Ratcliffe, Liza P Moorman, Matthew T Clark, Katy N Krahn, Oliver J Monfredi, Susan Hamil, Gholamreza Yousefvand, J Randall Moorman, Jamieson M Bourque

**Affiliations:** 1 University of Virginia Charlottesville, VA United States; 2 AMP3D Charlottesville, VA United States

**Keywords:** predictive analytics monitoring, AI, randomized controlled trial, risk estimation, clinical deterioration, visual analytics, artificial intelligence, monitoring, risk, prediction, impact, cardiology, acute care

## Abstract

**Background:**

Patients in acute care wards who deteriorate and are emergently transferred to intensive care units (ICUs) have poor outcomes. Early identification of patients who are decompensating might allow for earlier clinical intervention and reduced morbidity and mortality. Advances in bedside continuous predictive analytics monitoring (ie, artificial intelligence [AI]–based risk prediction) have made complex data easily available to health care providers and have provided early warning of potentially catastrophic clinical events. We present a dynamic, visual, predictive analytics monitoring tool that integrates real-time bedside telemetric physiologic data into robust clinical models to estimate and communicate risk of imminent events. This tool, Continuous Monitoring of Event Trajectories (CoMET), has been shown in retrospective observational studies to predict clinical decompensation on the acute care ward. There is a need to more definitively study this advanced predictive analytics or AI monitoring system in a prospective, randomized controlled, clinical trial.

**Objective:**

The goal of this trial is to determine the impact of an AI-based visual risk analytic, CoMET, on improving patient outcomes related to clinical deterioration, response time to proactive clinical action, and costs to the health care system.

**Methods:**

We propose a cluster randomized controlled trial to test the impact of using the CoMET display in an acute care cardiology and cardiothoracic surgery hospital floor. The number of admissions to a room undergoing cluster randomization was estimated to be 10,424 over the 20-month study period. Cluster randomization based on bed number will occur every 2 months. The intervention cluster will have the CoMET score displayed (along with standard of care), while the usual care group will receive standard of care only.

**Results:**

The primary outcome will be hours free from events of clinical deterioration. Hours of acute clinical events are defined as time when one or more of the following occur: emergent ICU transfer, emergent surgery prior to ICU transfer, cardiac arrest prior to ICU transfer, emergent intubation, or death. The clinical trial began randomization in January 2021.

**Conclusions:**

Very few AI-based health analytics have been translated from algorithm to real-world use. This study will use robust, prospective, randomized controlled, clinical trial methodology to assess the effectiveness of an advanced AI predictive analytics monitoring system in incorporating real-time telemetric data for identifying clinical deterioration on acute care wards. This analysis will strengthen the ability of health care organizations to evolve as learning health systems, in which bioinformatics data are applied to improve patient outcomes by incorporating AI into knowledge tools that are successfully integrated into clinical practice by health care providers.

**Trial Registration:**

ClinicalTrials.gov NCT04359641; https://clinicaltrials.gov/ct2/show/NCT04359641

**International Registered Report Identifier (IRRID):**

DERR1-10.2196/29631

## Introduction

Patients in acute care wards who deteriorate and are emergently transferred to the intensive care unit (ICU) have poor outcomes [1-6]. Subacute illnesses, such as sepsis, hemorrhage, and respiratory decompensation, occur in 5% or more patients and result in significant increases in length of stay and mortality [7]. Although potentially catastrophic, these subacute illnesses can be mitigated or indeed avoided depending on timely decisions made by the care team. Simply paying greater attention to patients who may be deteriorating from imminent changes in physiological status can allow for earlier clinical action and care to be appropriately escalated [8,9]. Informing clinicians of those patients at greater risk for otherwise unexpected clinical deterioration rather than waiting until after the common vital signs become noticeably abnormal and usual care alarms are triggered may provide a window for treatment that is more effective. Nurses’ worry or concern often precedes obvious deterioration in vital signs, suggesting that quantification of “concern” might lead to detection at an early stage when intervention may be more effective [10].

Advances in continuous bedside monitoring technology make a wealth of data available to assist evaluation by health care providers [11]. These data form the foundation for computational algorithms (artificial intelligence [AI]) that integrate real-time telemetric physiologic data to provide early warning of potentially catastrophic clinical events, including sepsis, emergent intubation, hemorrhage, and other events of clinical deterioration [12,13]. We have found subtle physiologic signatures of illness that are detectable through advanced mathematical analysis of cardiorespiratory dynamics trends up to 24 hours in advance of overt clinical deterioration [12,14-17]. Continuous predictive analytics monitoring involves advanced mathematical analysis of data from a variety of inputs (including parameters derived from telemetric monitoring) into an estimate of fold increase of risk that clinicians can observe in real time in a streaming environment [18,19].

In the neonatal setting, Griffin and Moorman [20] detected abnormal heart rate characteristics in the hours preceding a clinical diagnosis of sepsis and developed methods to process, characterize, and synthesize unprocessed cardiorespiratory monitoring data into a computational model that produced an estimation of risk. When these risk scores were visually displayed in the intervention arm of a multicenter randomized clinical trial of over 3000 very low birth weight neonates, there was a 20% decrease in mortality [21,22]. Thus, we know that early detection of deterioration improves outcomes, cardiorespiratory monitoring data are readily available, and trend-based models adding continuous monitoring outperform static vital sign models. We do not know, however, if displaying these visual AI risk scores can impact outcomes in adult patients of a variety of ages and historical comorbidities, who are susceptible to an array of adverse outcomes, as it does the outcomes in premature infants susceptible to sepsis.

**Figure 1:**
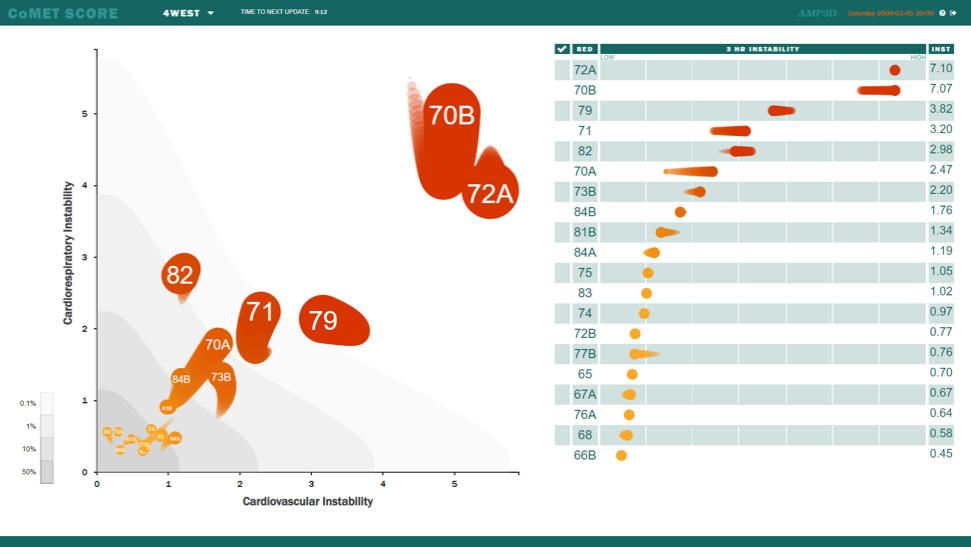
CoMET artificial intelligence–based visual risk analytic. CoMET: Continuous Monitoring of Event Trajectories.

Here, a cluster randomized controlled trial (NCT04359641) will test the use of Continuous Monitoring of Event Trajectories (CoMET; Figure 1), an AI-based visual analytic that dynamically displays risk estimates every 15 minutes for multiple adverse outcomes. CoMET uses continuous cardiorespiratory monitoring data and waveforms sampled every 2 seconds to perform mathematical measurements, such as measures of entropy and heart rate variability. R-R intervals and electrocardiogram-derived breathing rate were obtained from 200-Hz electrocardiogram waveforms; laboratory data and nurse-entered vital signs were obtained from the electronic medical records. These are used to derive an estimate of the fold increase in the risk of clinical deterioration. The models that have informed CoMET development have been described previously [7,12,18,23]. We expect that having access to a visual risk analytic for impending catastrophic outcomes will draw the clinicians’ attention to those patients who warrant early or extra consideration. For example, those patients with a higher risk score for short-term crises may be prioritized for assessment during daily rounds. This study will test the use of the CoMET display on patient outcomes, the time to proactive clinical action, and the associated costs to the health care system.

## Methods

### Overall Design

We will be conducting a parallel, cluster randomized controlled trial (RCT). All patients who are admitted to an 85-bed acute care cardiology medical-surgical floor will be enrolled in the study. In June 2020, institutional review board approval was granted for the Predictive Monitoring-Impact in Acute Care Cardiology Trial (PM-IMPACCT) along with waiver of informed consent, given the minimal risk nature of the study protocol. Clusters will be defined by room number, and upon enrollment, patients will be assigned to either intervention (CoMET display) or usual care (standard of care). The intervention will consist of CoMET display and charting in electronic medical records, mention of CoMET score on rounds, and standard of care cardiorespiratory monitoring when ordered. Meanwhile, usual care will consist of standard of care cardiorespiratory monitoring when ordered and no CoMET score displayed, charted, or mentioned on rounds. The study period will be 20 months. This report follows the SPIRIT (Standard Protocol Items: Recommendations for Interventional Trials) reporting guideline [24].

### Intervention Details

Data will be automatically collected electronically in real time for all patients (intervention and usual care) admitted to the randomized beds on the cardiology floor. The CoMET server is hosted within the hospital information technology environment and interfaces with the hospital interface engine to automatically gather clinical information, including numerical values in flow sheets, laboratory results, and continuous cardiorespiratory monitoring data [25]. For the intervention group only, the scores will then be displayed on monitors in real time and will be updated every 15 minutes.

Clinical research coordinators will routinely be present on inpatient rounding to bring up the web-based version of the CoMET display for the intervention arm and discuss relevant trends and answer questions about CoMET interpretation. Nurses will be asked to assess CoMET scores as part of routine vital sign collection. Every hour, the CoMET scores flow through directly to the electronic medical record vital sign flowsheet. Assessment protocol guidelines in response to CoMET scores on the intervention arm will suggest that if the CoMET score rises by 2 or more within a period of 3 hours, a nurse should assess the patient and consider alerting the nurse practitioner or resident physician caring for that patient. There is no direct clinical action or response mandated as a part of this RCT*.*

### Inclusion and Exclusion Criteria

All patients admitted to one of the 85 beds in 56 rooms on the acute care cardiology medical-surgical and cardiothoracic surgery floor (which encompasses a floor of the University of Virginia Medical Center and is further divided into 3 wards) will be enrolled in this study.

### Randomization Process

The room and bed in which a patient is placed is based on standard of care. The rooms are being cluster randomized to either intervention or usual care. The assignment of patients to rooms will not be influenced by the randomization status of the room.

There will be 14 clusters containing an average of 4 rooms (6 beds) each, with the aim of maintaining balance across wards and units (intermediate care unit, transplant, or neither). Clusters will be rerandomized every 2 months following a replicated Latin square design within each stratum to ensure balanced treatment assignment within each time period and over the length of the study. Randomization will be performed using the randomization inference package in R (The R Foundation for Statistical Computing). The randomization will be masked until it is implemented every 2 months. Postimplementation, the randomization will not masked or blinded because it will be clear to anyone in the hall or central station which beds have a CoMET score displayed and which do not. Future randomization assignments will only be known by the study statistician (SR) and those technical personnel implementing CoMET at the time that randomization changes (MC).

### Implementation and Education Considerations for Clinicians

All clinicians will be educated about CoMET to follow assessment guidelines in response to CoMET score. With consideration to alert fatigue, an increase of 2 or more within a period of 3 hours was selected as a guideline for assessment. In our retrospective cohort, a rise >2 units occurs 2 to 3 times per ward per day. On average then, a single nurse may experience 1 CoMET rise alert every 3 shifts. The clinicians will be provided education about what a score rising on either (or both) axes represents and the differential diagnoses to consider. This approach leaves the clinical context to that of the clinician and does not specify the specific diagnostics to order. The purpose of CoMET is to alert clinicians to prodromes or subtle physiological changes that precede overt clinical signs and draw attention to patients who may be in need of further attention, further clinical diagnostics, or escalation in care delivery. Clinicians may choose to draw a blood culture, initiate a rapid response team call, initiate oxygen therapy, or to do nothing but closely monitor or reassess the patient in an hour. Because CoMET models are trained on multiple events of clinical deterioration, clinicians understand the context of the CoMET score within the larger patient trajectory. There will be continual engagement and re-education throughout the entire study period across all clinician groups.

### Study Outcomes and Statistical Analysis

#### Primary Hypothesis

The primary hypothesis is that display of predictive analytics monitoring on acute care cardiology wards increases the number of hours free from clinical deterioration.

The primary outcome is the number of hours free from clinical deterioration (acute clinical events) within 21 days of admission. Hours of acute clinical events are defined as the time when one or more of the variables found in Textbox 1 occur. A maximum score will be 21 event-free days (504 hours). Patients who are discharged from the hospital prior to 21 days without an event will be counted as having 21 event-free days (504 hours). Patients who die during the admission will be counted as having 0 event-free days (0 hours). As an example, a patient who has an emergent ICU transfer on hour 26 of their admission will have 25 event-free hours. Patients will be censored (with no event observed) at the time of nonemergent ICU transfer (ie, for planned bronchoscopy), nonemergent surgery transfer, or other transfer (change in bed assignment) because a change in bed assignment can result in a change in randomization allocation (as carry over could occur in patients who have a display and then become “undisplayed”). We will conduct a randomized (formerly called “intent-to-treat”) analysis, in which all available data on all randomized participants are included for the primary end point comparison between intervention and usual care.

For the primary outcome (hours free of an acute clinical event), the primary analysis will employ a generalized estimating equation (GEE) with Poisson link. This model will be able to handle the multiple levels of correlation in the study design—patients within a cluster and potentially different intervention arms within a patient who has an event and then returns to the ward—as well as differences in the amount of time at risk for each patient that is censored.

**Study outcomes by hypotheses.**

**Hypothesis 1 (primary study outcome)**

1. Hours free from the following:

- An emergent intensive care unit (ICU) transfer (emergent defined as urgent, unplanned) and ICU stay
- Emergent intubation (emergent is defined by clinician’s notes as a nonplanned procedure)
- Cardiac arrest, if prior to ICU transfer
- Death
- Emergent surgery prior to an ICU transfer (emergent is defined by clinician’s notes as a nonplanned procedure)

**Hypotheses 2 and 4**

1. Proportions of the following individual events at any point in the hospital stay after admission to the fourth floor:

- Emergent ICU transfer (emergent defined as urgent, unplanned)
- Emergent intubation (emergent is defined by clinician’s notes as a nonplanned procedure)
- 3 units or more of blood ordered in 24 hours
- inotropes or vasopressor use
- Shock requiring inotropes or vasopressors
- Sepsis (sepsis-2 definition)
- Septic shock requiring inotropes or pressors (defined by a combination of the 2 above criteria)
- Cardiac arrest
- Death
- Diuretic drip indicating congestive heart failure escalation
- Inotropes or pressors for heart failure
- Emergent surgery prior to ICU transfer (emergent is defined by clinician’s notes as a nonplanned procedure)

2. Hospital length of stay

3. In patients who are never transferred to the ICU, the length of stay on the acute care floor

4. ICU length of stay

5. Proportion with readmission to hospital within 72 hours postdischarge

6. In patients who meet the sepsis-2 criteria, the proportion of shock (ie, hypotension requiring inotropes or pressors), and death

7. Number of days on intravenous antibiotics

8. Number of days on intravenous anti-infectives

9. Total duration of mechanical intubation (emergent and nonemergent)

10. Impact of sex on outcome

**Hypothesis 2**

1. Time of emergent intubation post-ICU transfer (emergent is defined by clinician’s notes as a nonplanned procedure)

2. Time of the first order post-ICU transfer for transfusion of 3 units or more of blood ordered within 24 hours

3. Time of first order post-ICU transfer of intravenous inotropes or pressors administered for shock (including septic shock)

4. Time of cardiac arrest post-ICU transfer

5. Time of congestive heart failure escalation, defined by the time of first order for diuretic drip, time of first order for continuous venovenous hemodialysis, or time of dialysis initiation

6. Time of death post-ICU transfer

7. “Infinite” event-free survival, defined as discharge from the ICU without an event

**Hypotheses 3 and 4**

1. Time to the first order for transfusion of 3 units or more of blood ordered within 24 hours

2. Time to first order for blood or urine culture obtained for suspicion of infection

3. Time to first order for lactate drawn

4. Time to first order for anti-infectives given for suspicion of infection

5. Time to first order for fluid resuscitation given for suspicion of shock

6. Time to rapid response team medical emergency team call initiation

7. Time to first order for intravenous inotropes or vasopressors administered

#### Secondary Hypothesis

The secondary hypothesis is that display of predictive analytics monitoring on acute care cardiology wards increases the number of hours free of clinical deterioration among those emergently transferred to the ICU.

We will use the Kaplan-Meier method or Cox proportional hazards regression curve to show post-ICU transfer event-free survival and hours free of the events listed in Textbox 1. The analysis comparison will employ a 1-sided clustered log-rank test (survival analysis) assuming proportional hazard rates.

For secondary outcomes measured as proportions (ie, yes-or-no outcomes) we will use GEEs with logit link and binomial distribution, and use negative binomial distribution for rare events. GEEs will allow the correct modeling of the clustering design and estimation of the intracluster correlation.

#### Tertiary Hypothesis

The tertiary hypothesis is that the display of predictive analytics monitoring shortens time to proactive clinical response. We will quantify hours to proactive clinical response using variables found in Textbox 1.

We will use a Cox proportional hazards regression curve to determine differences in response time between intervention and usual care. We will also collect and analyze end points to evaluate clinician response and elements of situational awareness, including the number of alerts observed (per patient days) and clinical actions chosen (both individual actions or team/communication actions). By characterizing the clinical actions, we will be able to more fully understand the outcomes of dynamic decision making in the context of a predictive analytic and future state of disease. We will also be able to assess fidelity to the clinical guidelines that were provided to clinicians as a component of the optimized alert strategy.

#### Quaternary Hypothesis

The quaternary hypothesis is that the display of predictive analytics monitoring reduces costs to the health system.

We will determine whether the display of predictive analytics is cost-effective to the health care system. Collection of end points to understand trade-offs and potential burdens, consequences, and advantages for this platform from the clinician perspective is critical and presents novel knowledge. Additionally, we can form an estimate for not only the cost of intervening but also the overall costs of implementing a predictive analytics or AI monitoring platform within a health system using multiple criteria (Table 1).

We will conduct an economic cost-effectiveness analysis comparing the clinical outcomes and associated cost of care between the intervention and usual care group. We will use a Markov decision tree and Monte Carlo simulation to examine the impact of CoMET compared with usual care from a health system perspective. We will measure incremental effect as the in-hospital mortality difference and the number of quality-adjusted life years gained discounted at a 3% annual rate as per the guidelines of the Public Health Service Panel on Cost-Effectiveness in Health and Medicine. We will measure differences in hospital costs based on internal data warehouse cost data weighted by the Centers for Medicare & Medicaid Services Cost Report cost-to-charge ratio [26].

**Table 1:** Elements included in health system decision analysis for artificial intelligence implementation.

Element	Example
ROI^a^	ROI = profit or cost, inclusive of costs related to clinical deterioration cases averted and costs related to unnecessary intervention (ie, false positives)
Capacity changes	C-score = (delta LOS^b^ in days x patients per week) + (added bed days/LOS of unit in days)
Staff training times	Average time to complete virtual cases per user group
Implementation costs	Costs associated with staff training or use of case-based approach
Impact on workforce	Number of false alarms as a unit of registered nurse time

^a^ROI: return on investment.

^b^LOS: length of stay.

#### Outcome Ascertainment and Event Adjudication

Numerical and categorical data (lab values, timestamps, room transfers, etc) will be extracted in bulk from the University of Virginia clinical data warehouse and uploaded into a Research Electronic Data Capture (REDCap) database for outcome analyses. For nonautomated outcome elements, such as suspicion of infection, emergent status of intubations, surgeries or transfers, suspicion of shock, actual shock, indications for vasopressors, and others, protocol-trained clinical research coordinators (consisting of registered nurses or medical doctors) will individually review the electronic medical records of case and control participants.

#### Sample Size, Power Analysis, and Attrition

We expect 11% of participants to drop out of the primary outcome because they will be in a room that switches the randomization arm at the time when rooms are rerandomized or because they will transfer rooms during their admission to a room of the opposite randomization or to a nonstudy room. We have accounted for this attrition in clinical trial planning and sample size considerations. The number of admissions to a room which will undergo cluster randomization is estimated to be 10,424. The study type 1 error rate will be set to 5% and the type II error rate to 20% (80% power). Additionally, an interim analysis will prespecified after half of the participants have completed their primary outcomes assessment. The interim analysis will use an O’Brien-Fleming spending function, giving nominal α values of .003 (interim) and .047 (final) for the 2 analyses. Should the primary outcome cross a stopping boundary at the interim analysis, the data safety monitoring board (DSMB) will have the option of stopping the study early.

## Results

### Trial Status

Prior to the study commencing, we were able to complete several key steps of the clinical trial planning, setting a solid foundation for the RCT. Specifically, we partnered with clinical and administrative leadership within the health system heart and vascular clinical service lines, we enlisted 12 nursing and physician partners who will serve as Super Users and CoMET champions, we conducted over 50 education sessions with nursing and physician clinical users (reaching over 120 clinicians to date) and have made 7 online educational activities available on the learning management system for staff at large, we obtained institutional review board and DSMB approval, we completed internal validation on CoMET (displays not turned on to clinical users), we refined clinical nursing and nurse practitioner or physician response guidelines through feedback from clinical stakeholders and nurses from the rapid response team, we completed the REDCap database and integration with clinical data warehouse (SQL queries written and validated), and DSMB members and chair were selected (9 members in total) and have met twice thus far. Randomization began January 4, 2021, and we anticipate the study taking 20 months of enrollment to complete.

## Discussion

We are evoking methodological approaches that have immersive properties (ie, clinician stakeholder engagement, immersive education, economic perspectives) and that offer a framework for scalability and long-term adoption. Additionally, we are taking on the task of conducting simultaneous evaluations of various populations within the acute care cardiology wards (surgical and medical patients with a variety of diagnoses). Even within a single hospital, each clinical unit will have varying environmental contexts of care, varying needs of the patient populations, and differing priorities of point-of-care clinicians, and we are including 3 distinct wards. In seeking to understand how to optimize a predictive analytic and effectively implement it within the context of a learning health system, we can learn how similar processes can be applied to nearly any predictive analytic and setting. Thus, we propose to focus on the process of testing these strategies through a randomized control trial and seek to disseminate consensus guidelines and provide expertise that can be applied to other analytics and a variety of care settings (a scalable approach). We anticipate a multicenter study following this single-center RCT.

We have extensive prior experience in the use of predictive analytics in the neonatal ICU and surgical-trauma ICU [11,23,27] through immersive qualitative approaches. We have learned that the clinicians prefer an open response protocol that relies on their expertise with some suggested actions without being too restrictive or adding additional burden to the system. These principles are also central to implementation science and pragmatic design perspectives. Further, our prior experience with the neonatal HeRO score [28] and the use of CoMET in a pre-post study in the surgical-trauma ICU [23] suggests that patient trajectory is of great use to practicing clinicians when the CoMET score is placed in the context of the overall clinical status. We have previously studied the role of alert thresholds using data from these hospital units and have optimized the sensitivity-versus-positive-predictive-value trade-off of establishing an alert threshold [29].

Very few AI-based analytics have been translated from algorithm to real-world use in complex health care settings. We believe that AI systems need to be developed and tested with end users in mind. Further, we think testing them in an RCT framework will allow for the assessment of efficacy and trade-offs within the system. Early identification of subtly worsening patients might allow for earlier clinical action and lead to reduced morbidity and mortality. The future of acute hospital care includes monitoring systems that integrate data streams of rapidly changing clinical information to estimate and communicate risk of imminent events. Too much data are collected and then discarded or ignored by the vast majority of hospital systems, representing an often crucial missed opportunity to optimize the care of patients. The proposed approach will facilitate a paradigm shift in care from reactive to proactive by exploiting all the data that are freely available in the majority of patients, using this to predict risk of adverse events and allowing clinicians to act early to promote optimal patient trajectories.

## Abbreviations

AI: artificial intelligence
DSMB: data safety monitoring board
GEE: generalized estimating equation
ICU: intensive care unit
PM-IMPACCT: Predictive Monitoring-Impact in Acute Care Cardiology Trial
RCT: randomized controlled trial
REDCap: Research Electronic Data Capture
SPIRIT: Standard Protocol Items: Recommendations for Interventional Trials
